# Hyperglycemia Is Associated With Computed Tomography Perfusion Core Volume Underestimation in Patients With Acute Ischemic Stroke With Large‐Vessel Occlusion

**DOI:** 10.1161/SVIN.123.001278

**Published:** 2024-04-13

**Authors:** Arash Niktabe, Juan Carlos Martinez‐Gutierrez, Sergio Salazar‐Marioni, Rania Abdelkhaleq, Juan Carlos Rodriguez Quintero, Jerome A. Jeevarajan, Muhammad Bilal Tariq, Ananya S. Iyyangar, Hussain M. Azeem, Anjan Nagesh Ballekere, Ngoc Mai Le, Louise D. McCullough, Sunil A. Sheth, Youngran Kim

**Affiliations:** ^1^ Department of Neurology UTHealth McGovern Medical School Houston TX; ^2^ Department of Neurosurgery UTHealth McGovern Medical School Houston TX; ^3^ Department of Management, Policy, and Community Health UTHealth School of Public Health Houston TX

**Keywords:** acute ischemic stroke, blood glucose, computed tomography perfusion, infarct core, large‐vessel occlusion

## Abstract

**Background:**

Computed tomography perfusion (CTP) predictions of infarct core play an important role in the determination of treatment eligibility in large‐vessel occlusion acute ischemic stroke. Prior studies have demonstrated that blood glucose can affect cerebral blood flow. Here, we examine the influence of acute and chronic hyperglycemia on CTP estimations of infarct core.

**Methods:**

From our prospectively collected multicenter observational cohort, we identified patients with large‐vessel occlusion acute ischemic stroke who underwent CTP with RAPID (IschemaView, Stanford, CA) postprocessing, followed by endovascular therapy with substantial reperfusion (Thrombolysis in Cerebral Infarction 2b–3) within 90 minutes, and final infarct volume determination by magnetic resonance imaging 48 to 72 hours posttreatment. Core volume overestimations and underestimations were defined as a difference of at least 20 mL between CTP‐RAPID predicted infarct core and Diffusion Weighted Imaging (DWI) final infarct volume. Primary outcome was the association of presentation glucose and hemoglobin A1c (HgbA1c) with underestimation of core volume and was measured using multivariable logistic regression adjusted for comorbidities and presentation characteristics. Secondary outcomes included frequency of overestimation of infarct core.

**Results:**

Among 256 patients meeting inclusion criteria, median age was 67 (interquartile range [IQR], 57–77) years, 51.6% were women, and 132 (51.6%) and 93 (36.3%) had elevated presentation glucose and elevated HgbA1c, respectively. Median CTP‐predicted core was 6 mL (IQR, 0–30 mL), median DWI final infarct volume was 14 mL (IQR, 6‐43 mL), and median difference was 12 mL (IQR, 5–35 mL). Twenty‐eight (10.9%) patients had infarct core overestimation and 68 (26.6%) had underestimation. Compared with those with no underestimation, patients with underestimation had elevated blood glucose (median, 119 [IQR, 103–155] versus 138 [IQR, 117–195] mg/dL; *P* = 0.002) and HgbA1c (median, 5.80% [IQR, 5.40–6.40] versus 6.40% [IQR, 5.50–7.90]; *P* = 0.009). In multivariable analysis, underestimation was independently associated with elevated glucose (adjusted odds ratio [OR], 2.10; *P* = 0.038) and HgbA1c (adjusted OR, 2.37; *P* = 0.012). Overestimation was associated with lower presentation blood glucose (median, 109 [IQR, 99–132] in overestimation versus 127 [IQR, 107–172] mg/dL in no overestimation; *P* = 0.003) and HgbA1c (5.6%[IQR 5.1–6.2] in overestimation versus 5.90%[IQR, 5.50–6.70] in no overestimation; *P* = 0.012).

**Conclusions:**

Acute and chronic hyperglycemia were strongly associated with CTP underestimation in patients with large‐vessel occlusion acute ischemic stroke undergoing endovascular therapy. Glycemic state should be considered when interpreting CTP findings in patients with large‐vessel occlusion acute ischemic stroke.

## Graphical abstract

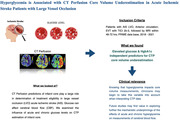

**Nonstandard Abbreviations and Acronyms**
AIS: acute ischemic stroke
CBF: cerebral blood flow
CTP: computed tomography perfusion
EVT: endovascular therapy
FIV: final infarct volume
LVO: large‐vessel occlusion

**Clinical Perspective**

- Hyperglycemia, particularly chronic state, shows strong association with computed tomography perfusion underestimation in patients with large‐vessel occlusion acute ischemic stroke undergoing endovascular therapy.
- Our margin to define underestimation is likely much larger than any acceptable error of measurement. Clinicians may begin to take glycemic state into account when interpreting computed tomography perfusion data.
- Future studies may focus on further uncovering mechanistic effects of hyperglycemia on measurements of cerebral blood flow.

Imaging evaluations play a central role in patient selection for endovascular therapy (EVT) in large‐vessel occlusion (LVO) acute ischemic stroke (AIS). One of the key elements of the imaging evaluation is an estimation of the size and location of irreversibly damaged tissue, or ischemic core. Computed tomography perfusion (CTP) is a technique that has been used for this purpose in many EVT randomized clinical trials.^1^, ^2^, ^3^, ^4^, ^5^, ^6^, ^7^ Accurate estimation of core volume is crucial for clinical decision‐making to determine the eligibility of EVT. Overestimating the core volume may potentially result in withholding treatment, whereas significant underestimation can lead to patients undergoing a procedure with reduced likelihood of benefit and an increased risk. Although CTP is commonly used in clinical practice today, the accuracy of CTP infarct core predictions remains incompletely characterized.

CTP infarct core estimations rely on asymmetry in cerebral blood flow (CBF) or volume between the 2 hemispheres. Prior studies have suggested that acute and chronic hyperglycemia can decrease global and regional CBF.^8^, ^9^, ^10^ We sought to examine the relationship between presentation glucose and hemoglobin A1c (HgbA1c) on CTP core predictions. We hypothesize that acute and chronic hyperglycemia are associated with increased CTP core/final infarct volume (FIV) measurement inaccuracies for patients undergoing EVT for LVO AIS.

## Methods

### Study Design and Participants

The data used in this study are available for collaborative groups, provided there is approval from the institutional review boards and data sharing agreements are in place. This study followed the Strengthening the Reporting of Observational Studies in Epidemiology reporting guideline, and the study protocol was reviewed and approved by the Institutional Review Board at The UTHealth Houston.

From our prospectively collected multicenter observational cohort study, PRIME (Practical Implementation of Mechanical Thrombectomy), we identified consecutive patients with AIS from 11 certified stroke centers (4 comprehensive stroke centers and 7 primary stroke centers) in a large metropolitan area. For this study, we included patients who were aged ≥18 years, diagnosed with anterior circulation LVO AIS, and underwent EVT from January 2018 to March 2021. Anterior circulation LVO was defined as occlusion of the intracranial internal carotid artery, M1 to M2 portions of the middle cerebral artery, or A1 anterior cerebral artery. Patients were excluded if they did not achieve substantial reperfusion, defined as Thrombolysis in Cerebral Infarction grade 2b to 3, or did not have both CTP imaging at presentation and magnetic resonance imaging (MRI) within 48 to 72 hours following reperfusion.

### Measurements

Blood glucose and HbA1c levels were measured at the time of admission, and they were dichotomized on the basis of receiver operating characteristic (ROC) curve analysis, described in the Statistical Analysis section (Figure S1). CTP postprocessing was performed using RAPID software (iSchemaView, Palo Alto, CA) with default parameters for postprocessing and defining core as the volume of CBF <30% (relative Cerebral Blood Volume, <30%) relative to the contralateral hemisphere.^11^ FIV was measured using the previously validated ABC/2 ellipsoid volume estimation calculations from diffusion‐weighted MRI 48 to 72 hours after successful reperfusion.^12^, ^13^, ^14^ Accuracy of CTP‐predicted infarct core was determined by comparing this volume against FIV. CTP estimation inaccuracy was categorized as either overestimation or underestimation, which were defined as CTP‐predicted core volumes of at least 20 mL greater or less than the FIV, respectively. This approach of using postreperfusion imaging as a ground truth to evaluate the accuracy of infarct core predictions on presentation imaging in patients with rapid reperfusion is consistent with prior publications.^15^ Stroke severity was measured using the National Institutes of Health Stroke Scale and trichotomized (0–15, 16–20, and 21–42). Early time window was defined as presentation within 6 hours from onset or last known well time.

### Outcomes

The primary outcome was the association between presenting blood glucose and HgbA1c with CTP underestimation. Secondary outcomes included the association between presenting blood glucose and HgbA1c with CTP overestimation.

### Statistical Analysis

The optimal thresholds of presentation blood glucose level and HgbA1c to predict underestimation were determined by ROC curve analysis using the nonparametric method suggested by DeLong et al.^16^ The optimal cutoff value was selected as the point on the ROC curve minimizing the Euclidean distance between the ROC curve and the (0,1) point, an ideal point representing 0 false positives and perfect sensitivity.^17^ These optimal cutoff points were then used to create dichotomized values for blood glucose and HgbA1c. In sensitivity analyses, we also used cutoffs for hyperglycemia and elevated HgbA1c, defined by the American Diabetes Association, which are blood glucose >200 mg/dL and HgbA1c >6.5%. Univariable analyses to assess the associations between presentation characteristics and underestimation were conducted, and crude odds ratios (ORs) with 95% CIs were reported from logistic regression independent associations of blood glucose and HgbA1c with underestimation and were assessed using multivariable logistic regression adjusting for age, sex, National Institutes of Health Stroke Scale score, occlusion location, and early window presentation. In sensitivity analysis, we reduced the threshold defining underestimation to 15 mL. In further sensitivity analysis, we used preexisting published cutoffs for diabetes (random blood glucose, >200 mg/dL) and diabetes (HgbA1c, >6.5%). Percentages and median interquartile ranges (IQRs) were reported for categorical and continuous variables, respectively, and Fisher exact tests and rank sum tests were used for significant differences. Significance levels were set at *P*<0.05 for 2‐tailed tests, and all analyses were performed using STATA 16.0 (StataCorp, College Station, TX).

## Results

Among 256 study participants who met inclusion criteria, 51.6% were women, median age was 67 (IQR, 57–77) years, median National Institutes of Health Stroke Scale score was 15 (IQR, 10–19), and median Alberta Stroke Program Early CT [Computed Tomography] Score was 8 (IQR, 7–10). Median CTP predicted core volume was 6 mL (IQR, 0–30 mL), and median FIV was 14 mL (IQR, 6–43 mL). Baseline characteristics between groups were similar. Patients with underestimation had significantly larger FIV median measurements compared with patients with no underestimation (73 [IQR, 47–135] versus 9 [IQR, 3–17] mL). A significantly higher percentage of patients with underestimation achieved a Thrombolysis in Cerebral Infarction 2b compared with those with no underestimation. The relative occurrence of hypertension and hyperlipidemia was higher in patients with underestimation compared with no underestimation, but the occurrence of congestive heart failure and coronary artery disease was proportionally higher in patients with no underestimation (Table 1). Compared with patients with no underestimation, patients with underestimation had greater admission blood glucose (median, 119 [IQR, 103–155] versus 138 [IQR, 117–195] mg/dL; *P* = 0.002) and greater HgbA1c (median, 5.80% [IQR, 5.40–6.40] versus 6.40% [IQR, 5.50–7.90]; *P* = 0.009) (Table 1). A greater proportion of patients with underestimation had glucose >200 mg/dL at presentation (13% versus 25%; *P*<0.026) and HgbA1c >6.5% (21% versus 41%; *P*<0.001).

**Table 1:** Baseline and Clinical Characteristics

Characteristic	Total (N = 256)	No underestimation (n = 188)	Underestimation by >20 mL (n = 68)	*P* value^*^
Age, median (IQR), y	67 (57–77)	67 (58–79)	66 (54–76)	0.16
Female sex, n (%)	132 (51.6)	103 (54.8)	29 (42.6)	0.09
Race and ethnicity				
Non‐Hispanic White	114 (44.5)	85 (45.2)	29 (42.6)	0.12
Non‐Hispanic Black	81 (31.6)	57 (30.3)	24 (35.3)	
Hispanic	42 (16.4)	28 (14.9)	14 (20.6)	
All other races	19 (7.4)	18 (9.6)	1 (1.5)	
NIHSS score, median (IQR)	15 (10–19)	14 (10–18)	16 (12–20)	0.1
Occlusion sites, n (%)				
Internal Carotid Artery‐intracranial	52 (20.3)	33 (17.6)	19 (27.9)	0.049
Middle Cerebral Artery‐M1	163 (63.7)	128 (68.1)	35 (51.5)	
Middle Cerebral Artery‐M2	41 (16.0)	27 (14.4)	14 (20.6)	
Imaging, median (IQR)				
ASPECTS	8 (7–10)	8 (7–10)	7 (6–10)	0.01
CTP estimated infarct core, mL	6 (0–30)	6 (0–24)	10 (0–47)	0.19
Final infarct volume, mL	14 (6–43)	9 (3–17)	73 (47–135)	<0.001
Absolute difference for CTP‐estimated infarct core and final infarct volume, mL	12 (5–35)	7 (3–14)	56 (31–93)	<0.001
Arrival blood glucose, median (IQR)				
Glucose, mg/dL	124 (105–165)	119 (103–155)	138 (117–195)	0.002
Hemoglobin A1c, %	5.85 (5.40–6.60)	5.80 (5.40–6.40)	6.40 (5.50–7.90)	0.009
Arrival blood pressure, median (IQR), mm Hg				
Systolic	154 (135–174)	154 (134–171)	156 (137–177)	0.68
Diastolic	83 (75–92)	82 (75–92)	84 (76–94)	0.59
Mean arterial pressure, median (IQR), mm Hg	107 (97–118)	106 (97–117)	108 (98–119)	0.62
Arrived within 6 h, n (%)	137 (53.5)	102 (54.3)	35 (51.5)	0.69
Intravenous tPA, n (%)	105 (41.0)	81 (43.1)	24 (35.3)	0.26
Final TICI, n (%)				
TICI 2b	74 (28.9)	42 (22.3)	32 (47.1)	<0.001
TICI 2c	22 (8.6)	18 (9.6)	4 (5.9)	
TICI 3	160 (62.5)	128 (68.1)	32 (47.1)	
Risk factors, n (%)				
Atrial fibrillation	52 (20.3)	38 (20.2)	14 (20.6)	0.95
Diabetes	89 (34.8)	61 (32.4)	28 (41.2)	0.20
Hypertension	157 (61.3)	112 (59.6)	45 (66.2)	0.34
Hyperlipidemia	94 (36.7)	67 (35.6)	27 (39.7)	0.55
Congestive heart failure	14 (5.5)	12 (6.4)	2 (2.9)	0.28
Coronary artery disease/MI	29 (11.3)	23 (12.2)	6 (8.8)	0.45

ASPECTS indicates Alberta Stroke Program Early CT [Computed Tomography] Score; CTP, CT perfusion; IQR, interquartile range; NIHSS, National Institutes of Health Stroke Scale; TICI, Thrombolysis in Cerebral Infarction; and tPA, tissue‐type plasminogen activator.

* Statistical significance was determined by χ^2^ test for categorical variables and Wilcoxon rank‐sum test for continuous variables.

The median difference between CTP‐predicted infarct core and FIV was 12 mL (IQR, 5–35 mL). A total of 96 (37.5%) patients had a substantial mismatch between CTP‐RAPID core predictions and FIV. In this cohort, 68 (26.6%) patients had underestimation of ≥20 mL and 28 (10.9%) had overestimation. Median difference between CTP‐predicted infarct core and FIV in the underestimation group was 56 mL (IQR, 31–93 mL).

The ROC curve estimated the optimal cutoff point for elevated presentation blood glucose at 124 mg/dL with the area under the curve of 0.620 and 6.3% for HgbA1c with the area under the curve of 0.622 (Figure S1). In univariable analysis, elevated blood glucose (OR, 2.71 [95% CI, 1.50–4.89]; *P* = 0.001) and HgbA1c (OR, 2.81 [95% CI, 1.59–4.98]; *P*<0.001) levels were associated with higher rates of underestimation. In multivariable analysis, we found both elevated blood glucose (adjusted OR, 2.10 [95% CI, 1.04–4.23]; *P* = 0.038) and HgbA1c (OR, 2.37 [95% CI, 1.21–4.63]; *P* = 0.012) are independently associated with underestimation. Women were less likely to have underestimation of their infarct core volume (adjusted OR, 0.49 [95% CI, 0.26–0.93]; *P* = 0.03) (Table 2). In the sensitivity analysis reducing the definition of underestimation to 15 mL, elevated glucose level was associated with underestimation but not statistically significant (adjusted OR, 1.72 [95% CI, 0.89–3.31]; *P* = 0.106), whereas elevated HgbA1c remained significantly associated with underestimation (adjusted OR, 2.43 [95% CI, 1.28–4.62]; *P* = 0.006) (Table S1). In additional sensitivity analysis, we repeated the multivariable regression using standard definitions for diabetes based on glucose >200 mg/dL and HgbA1c >6.5%. In this analysis, glucose elevated beyond 200 mg/dL was not associated with underestimation (OR, 1.4 [95% CI, 0.55–3.6]) but HgbA1c >6.5% was associated with underestimation (OR, 2.5 [95% CI, 1.1–5.6]).

**Table 2:** Factors Associated With Underestimation (Cutoff of 20 mL)

Factor	Odds ratio (95% CI)	*P* value	Adjusted odds ratio (95% CI)	*P* value
Age group, y				
18–54	1 (Reference)		1 (Reference)	
55–64	0.86 (0.38–1.94)	0.71	0.67 (0.27–1.67)	0.39
65–74	0.68 (0.31–1.50)	0.34	0.55 (0.23–1.30)	0.17
≥75	0.69 (0.32–1.47)	0.33	0.72 (0.31–1.67)	0.44
Sex				
Male	1 (Reference)			
Female	0.61 (0.35–1.07)	0.087	0.49 (0.26–0.93)	0.03
National Institutes of Health Stroke Scale				
0–15	1 (Reference)			
16–20	1.91 (1.02–3.56)	0.04	2.04 (1.02–4.09)	0.04
21–42	1.74 (0.80–3.79)	0.16	2.29 (0.96–5.44)	0.06
Occlusion site				
ICA‐intracranial	1 (Reference)			
MCA‐M1	0.47 (0.24–0.93)	0.03	0.48 (0.23–1.02)	0.06
MCA‐M2	0.90 (0.38–2.12)	0.81	1.30 (0.50–3.42)	0.59
Early window	0.89 (0.51–1.56)	0.69	0.84 (0.46–1.54)	0.56
Glucose ≥124 mg/dL	2.71 (1.50–4.89)	0.001	2.10 (1.04–4.23)	0.038
Hemoglobin A1c ≥6.3%	2.81 (1.59–4.98)	<0.001	2.37 (1.21–4.63)	0.012
Risk factors				
Atrial fibrillation	1.02 (0.51–2.03)	0.95		
Diabetes	1.46 (0.82–2.58)	0.20		
Hypertension	1.33 (0.74–2.37)	0.34		
Hyperlipidemia	1.19 (0.67–2.10)	0.55		
Congestive heart failure	0.44 (0.10–2.04)	0.30		
Coronary artery disease/MI	0.69 (0.27–1.79)	0.45		

MI indicates myocardial infarction.

The median difference between CTP‐predicted infarct core and FIV in patients with overestimation was 40 mL (IQR, 24–56 mL). Compared with patients with underestimation, patients with overestimation had lower blood glucose (median, 109 [IQR, 99–132] versus 126 [IQR, 107–172] mg/dL; *P*<0.003) and HgbA1c (median, 5.6% [IQR, 5.1–6.2] versus 5.9% [IQR, 5.5–6.7]; *P*<0.05). In logistic regression, presentation glucose was associated with likelihood of overestimation (OR, 0.40 [95% CI, 0.18–0.93]).

## Discussion

In this cohort of ≈250 patients with LVO AIS and successful reperfusion after EVT, ischemic core volume mismatch between RAPID CTP and FIV measured on MRI‐DWI occurred in ≈40% of patients, with 11% overestimation and 27% underestimation. Overestimation was more likely to occur in patients presenting in the early time window, consistent with prior studies.^18^, ^19^ Elevated glucose and HgbA1c at presentation were associated with a significant increase in the likelihood of underestimation. Here, we used ROC curve analysis to define the optimal cutoff points for blood glucose and HgbA1c to predict core underestimation. The current American Diabetes Association guideline defines random blood glucose and HgbA1c of 200 mL/dl and 6.5%, respectively, as cutoff values for diabetes. Thus, our cutoff point of 6.3% for HgbA1c was in fact in the upper limit for prediabetes (5.7%–6.4%), and nearly half of our subjects with underestimation met the HgbA1c cutoff for diabetes per American Diabetes Association guideline (≥6.5%).^20^

Patients with AIS who present with acute hyperglycemia or diabetes are more likely to have a less favorable clinical outcome and 90‐day mortality, regardless of recanalization method.^21^, ^22^ Other studies have also looked at hyperglycemia in the context of clinical outcome as an established predictor of infarct growth and a treatment effect modifier of mechanical thrombectomy.^23^, ^24^, ^25^ On the other hand, CTP estimation of the infarct core volume in patients with LVO AIS relies on relative reduction in blood flow compared with the unaffected reference hemisphere. Acute hyperglycemia has shown to decrease the CBF.^8^ Consequently, in a hyperglycemic state, the reference hemisphere leveraged by CTP is likely to be hypoperfused, leading to a smaller core detection. Patients with long‐standing diabetes usually have poor collaterals, which can further drive rapid progression of ischemic core.^26^, ^27^, ^28^ Thus, the infarct core can potentially enlarge before reperfusion, resulting in underestimation at the time of presentation. These findings are supported by our study here, where a higher percentage of patients with acutely elevated blood glucose levels and HgbA1c have CTP core volume underestimation when compared with the MRI‐DWI.

Patients presenting with acute stroke often have elevated blood glucose. Studies suggest that lactic acidosis, oxidative stress, activation of cell death pathways, and mitochondrial dysregulations may play a role in the way that hyperglycemia exacerbates ischemic brain injury.^29^ Elevated glucose levels have shown to be linked to decreased brain levels of high‐energy phosphates. This can disturb the cellular membrane ion homeostatic process that can potentially exacerbate ischemic injuries by making the brain more vulnerable.^30^ In addition, increased blood‐brain barrier permeability may also play a key role in how glucose may affect stroke patients.^31^ Chronic hyperglycemia, in particular, may impact CBF through microvasculature remodeling. Studies show that mean transient time increases on the nonischemic side in patients with poorly managed glucose. This has been attributed to increased number of intracranial capillaries and their resistance, which is likely attributable to the increased media/lumen ratio and stiffness.^32^ In addition, long‐standing hyperglycemia has shown to damage intracranial vasculatures through endothelial dysfunction. This happens through increased reactive oxygen species, disruption of endothelial phenotype transcription, and processes that disturb the natural reaction of the vessels to necessary hemodynamic adaptations.^33^

In our sensitivity analysis using the American Diabetes Association guideline, glucose levels >200 mg/dL were not significantly associated with CTP core volume underestimation. We believe that the fluctuating levels of glucose in an acute setting can potentially explain why the sensitivity analysis failed to show a significant association between glucose levels and CTP core volume underestimation; as such, acute elevation may be a response to the stroke itself.^34^, ^35^ However, HgbA1c levels >6.5 did show a significant association with underestimation in CTP measurements, which shows that the chronic hyperglycemia could potentially interfere with CTP‐based interpretations of CBF. The occurrence of congestive heart failure and coronary artery disease was higher in patients with no underestimation. It is possible that in these patients, altered cardiac function results in deviations of the time‐density curve from which perfusion parameters are calculated, which would be an association for future studies to investigate.

To date, early time window is the best described variable associated with CTP inaccuracy.^36^, ^37^ We saw that patients who presented in the early time window (<6 hours) were more likely to have CTP core volume overestimation, but not underestimation. Because CTP measurements are a snapshot of blood flow to the brain in time, we can anticipate that areas defined as core of ischemia by a CBF of ≤30% can potentially be salvageable if a successful reperfusion is achieved. It seems that our cohort is consistent with such anticipated outcome. We found that in patients who presented in the early time window and achieved successful reperfusion, their core volume had been overestimated on CTP. This finding also reiterates the significance of door‐to‐needle time for patients with AIS secondary to LVO.

One limitation in our study is that infarct growth could have occurred after the CTP and before reperfusion. If this were the case, patients would appear to be “underestimated.” However, our method of using patients with rapid endovascular reperfusion has been used by prior studies.^38^ Although it may be the case that such expansions occur more commonly in patients with acute or chronic hyperglycemia, as they may have worse collaterals, this difference is still clinically relevant. The more rapid expansion resulting in underestimation would still be an important aspect to be aware of when evaluating CTP results in these patients. Furthermore, we intentionally chose a large threshold of 20 mL to define underestimation, including cases in which there was substantial disparity between CTP predictions and FIV measurements. Another limitation to our study is the inherent inaccuracies of the CTP imaging modality. This margin of error makes it difficult to ascertain if acute/chronic hyperglycemia is contributing to the changes in core size from CTP to MRI. However, we chose a large margin of 20 mL as this margin is likely much larger than any acceptable inherent error of measurement. Furthermore, although we limited the cohort to patients with Thrombolysis in Cerebral Infarction 2b/3 reperfusion to limit the likelihood of infarct growth between initial and final infarct imaging, this level of reperfusion is attained in nearly 90% of modern‐day EVT cohorts, and as such these findings apply to the vast majority of patients being considered for EVT.^39^

In conclusion, hyperglycemia and elevated HgbA1c at the time of presentation were strongly associated with CTP infarct core volume underestimations for patients with LVO AIS undergoing EVT. Part of the role of clinicians is to know the limitations of their tools and the extent to which we can rely on certain diagnostic modalities. Knowing that hyperglycemia impacts core volume measurements, clinicians may begin to take this variable into account when interpreting CTP data, and further studies may find value in exploring further the mechanistic underpinnings of the effects of acute and chronic hyperglycemia on measurements of CBF.

## Sources of Funding

Dr Sheth reports funding from the National Institutes of Health (R01NS121154). The funding organization had no role in the design and conduct of the study; collection, management, analysis, and interpretation of the data; preparation, review, or approval of the manuscript; and decision to submit the manuscript for publication.

## Disclosures

Dr Sheth reports other grant support from the National Institutes of Health (R01NS121154) as well as Viz.AI for unrelated projects. He also reports consulting fees from Viz.AI, Penumbra, and Imperative Care outside of the submitted work. Sunil Sheth is an Associate Editor for S:VIN and was not involved in the handling or final disposition of this article. Disclosures provided by in compliance with American Heart Association's annual Journal Editor Disclosure Questionnaire are available at https://www.ahajournals.org/editor-coi-disclosures. Authors Niktabe, Martinez‐Gutierrez, Salazar‐Marioni, Abdelkhaleq, Rodriguez Quintero, Jeevarajan, Tariq, Iyyangar, Zeem, Ballekere, Le, McCullough, and Kim report no relevant disclosures.

## Supporting information

Figure S1
Table S1
