# Supplementary material for: Hyperglycemia Is Associated With Computed Tomography Perfusion Core Volume Underestimation in Patients With Acute Ischemic Stroke With Large‐Vessel Occlusion
Source: Stroke Vasc Interv Neurol. 2024 Apr 13;4(4):e001278. doi: 10.1161/SVIN.123.001278 (PMC11460650; doi:10.1161/SVIN.123.001278)
Supplement: Supplementary file 1 — Figure S1 Table S1 [file SVI2-4-e001278-s001.pdf]

Supplemental Data

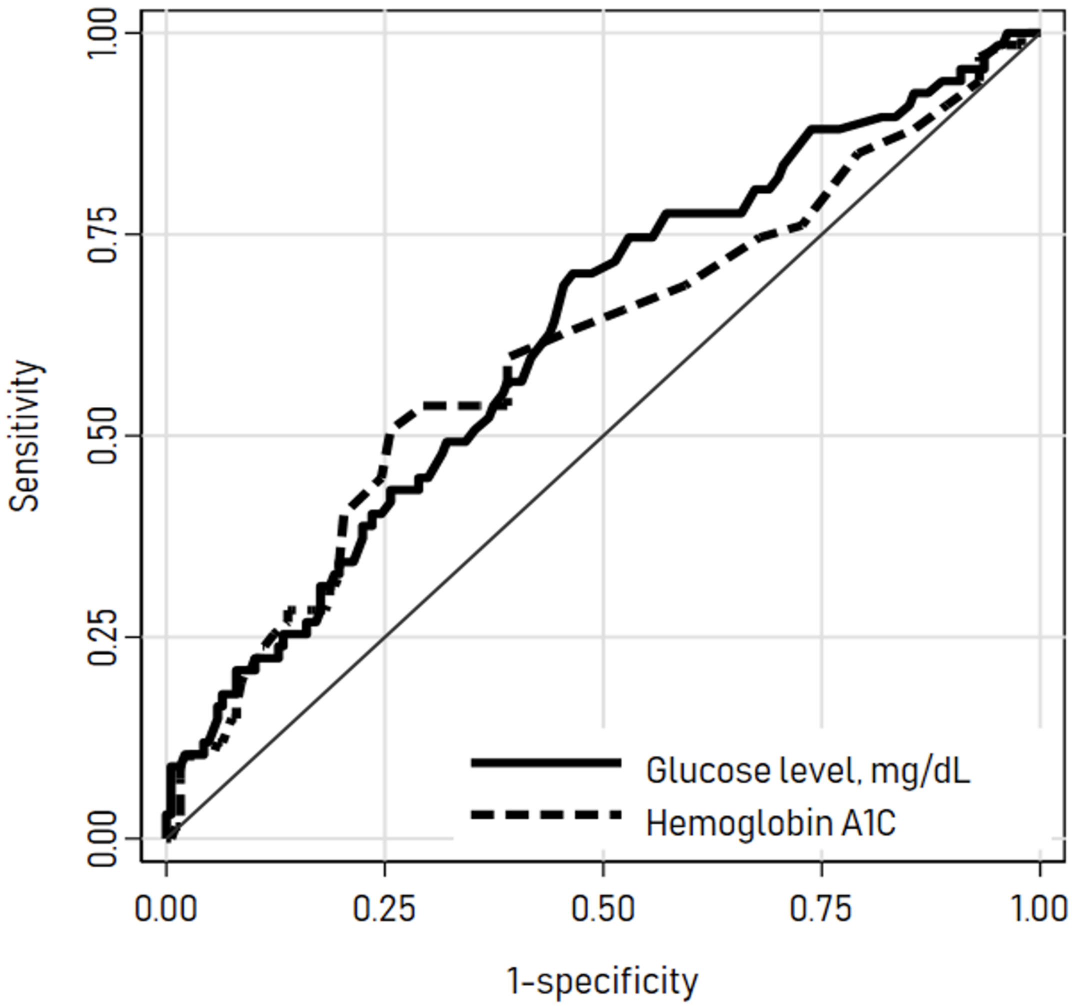

Supp. Fig. 1 The ROC curve examining the AUC for glucose levels and hemoglobin A1C in UE (Cutoff=20 mL). For glucose, empirical optimal cut-point was 124 with area under the curve (AUC) 0.62 and sensitivity and specificity at cut-point were 0.69 and 0.55 respectively**.** For hemoglobin A1c, empirical optimal cut-point was 6.3 with AUC 0.62 and sensitivity and specificity at cut-point were 0.54 and 0.71 respectively. C-statistics (area under curve, AUC) was not statistically different between glucose and A1C, p=0.55

Supplemental Table 1. Factors associated with underestimation (cutoff 15 mL)

	Odds ratios	p-value	aOdds ratios	p-value
**Age group**				
18-54	1 (reference)		1 (reference)	
55-64	0.80 (0.36 - 1.78)	0.59	0.61 (0.25 - 1.48)	0.28
65-74	0.73 (0.34 - 1.57)	0.42	0.59 (0.26 - 1.35)	0.21
75+	0.86 (0.42 - 1.76)	0.67	0.93 (0.42 - 2.07)	0.86
**Sex**				
Male	1 (reference)			
Female	0.55 (0.32 - 0.93)	0.027	0.41 (0.22 - 0.75)	0.00
**NIHSS**				
0-15	1 (reference)			
16-20	1.59 (0.88 - 2.89)	0.13	1.69 (0.87 - 3.27)	0.12
21-42	1.79 (0.86 - 3.74)	0.12	2.35 (1.03 - 5.33)	0.04
**Occlusion site**				
ICA-Intracranial	1 (reference)			
MCA-M1	0.66 (0.34 - 1.28)	0.22	0.67 (0.33 - 1.39)	0.29
MCA-M2	1.11 (0.48 - 2.59)	0.81	1.62 (0.63 - 4.18)	0.32
**Early window**	0.81 (0.48 - 1.38)	0.45	0.74 (0.41 - 1.31)	0.30
**Glucose>=124 mg/dL**	2.23 (1.29 - 3.85)	0.004	1.72 (0.89 - 3.31)	0.106
**Hemoglobin A1C >=6.3**	2.71 (1.57 - 4.68)	0.00	2.43 (1.28 - 4.62)	0.006
**Risk Factors**				
Atrial Fibrillation	1.09 (0.57 - 2.08)	0.80		
Diabetes	1.39 (0.81 - 2.41)	0.24		
Hypertension	1.26 (0.73 - 2.18)	0.42		
Hyperlipidemia	1.23 (0.71 - 2.11)	0.46		
Congestive Heart Failure	0.35 (0.08 - 1.60)	0.18		
Coronary Artery Disease/MI	0.54 (0.21 - 1.38)	0.20		
